# Forecasting Pedestrian Movements Using Recurrent Neural Networks: An Application of Crowd Monitoring Data

**DOI:** 10.3390/s19020382

**Published:** 2019-01-18

**Authors:** Dorine C. Duives, Guangxing Wang, Jiwon Kim

**Affiliations:** 1 Transport & Planning, Delft University of Technology, 2628 CN Delft, The Netherlands; 2 School of Civil Engineering, The University of Queensland, Brisbane St. Lucia, QLD 4072, Australia; guangxing.wang@uq.edu.au (G.W.); jiwon.kim@uq.edu.au (J.K.)

**Keywords:** deep learning algorithms, forecasting, pedestrian crowd movements, GPS trajectories, Recursive Neural Network (RNN) with Gated Recurrent Unit (GRU)

## Abstract

Currently, effective crowd management based on the information provided by crowd monitoring systems is difficult as this information comes in at the moment adverse crowd movements are already occurring. Up to this moment, very little forecasting techniques have been developed that predict crowd flows a longer time period ahead. Moreover, most contemporary state estimation methods apply demanding pre-processing steps, such as map-matching. The objective of this paper is to design, train and benchmark a data-driven procedure to forecast crowd movements, which can in real-time predict crowd movement. This procedure entails two steps. The first step comprises of a cell sequence derivation method that allows the representation of spatially continuous GPS traces in terms of discrete cell sequences. The second step entails the training of a Recursive Neural Network (RNN) with a Gated Recurrent Unit (GRU) and six benchmark models to forecast the next location of pedestrians. The RNN-GRU is found to outperform the other tested models. Some additional tests of the ability of the RNN-GRU to forecast illustrate that the RNN-GRU preserves its predictive power when a limited amount of data is used from the first few hours of a multi-day event and temporal information is incorporated in the cell sequences.

## 1. Introduction

Crowd monitoring systems are maturing rapidly due to the fast advancement of sensor technologies. Wi-Fi sensors can be used to determine the activity patterns of pedestrians (e.g., [1,2]) and measure the number of people in the vicinity of the Wi-Fi sensor (among other works [3]). Automated counting systems, such as real-time automatic video detection software in combination with cameras, radar and Lidar, are used to identify the amount of flow in certain corridors (e.g., [4,5]). GPS trackers provide insights into the complete moment patterns of subset of the moving crowd (e.g., [6]). Recently, combinations of sensor are used to create a comprehensive overview of the movement patterns at large-scale events [7].

Even though crowd monitoring systems already provide much-needed insights into the unfolding of crowd movement dynamics, effective crowd management based on the information provided by these systems is difficult as this information comes in at the moment the adverse crowd movements are already occurring. That is, crowd monitoring systems do not yet provide enough response time to pre-emptively determine the perspective for action and implement the necessary crowd management solutions. In order to improve the effectivity and efficiency of crowd management, a method to forecast crowd movements in real-time based on real-time crowd monitoring information is wanted.

Up to this moment, forecasting procedures to predict longer term crowd movements that do not rely on historic information regarding the crowds’ movements on previous days are limited. Most presented methods either use a very small prediction horizon, e.g., [8], or extrapolate the crowds’ movements patterns on previous days (e.g., [9]). In case of large-scale events, no historic information exists as the pedestrian infrastructure changes every year. Moreover, a forecasting horizon of at least 15 min is necessary in order to set-up and deploy the necessary intervention strategies. On the other hand, forecasting models with these two specific properties are often used to manage vehicular traffic. Both data-driven and model-driven solutions have been developed to forecast the flow of vehicles through large networks. The ‘older’ model-driven solutions use macroscopic models such as LWR and the cell transmission model. Data-driven solutions make use of smart filtering techniques (e.g., Kalman and Particle Filters) and machine learning techniques (e.g., AutoRegressive Integrated Moving Average (ARIMA) models, Markov Chain models and several types of neural networks, e.g., [10,11,12,13,14]). These procedures can possibly be adapted to also forecast pedestrian traffic flows.

The objective of this paper is to develop a forecasting method that can predict pedestrian movements at large-scale events in real-time which is fed using GPS traces. A promising data-driven urban vehicular traffic forecasting technique, namely that of [15], is adapted in order to predict pedestrian movements at large-scale events based on GPS traces. This forecasting technique comprises of two steps. The first step comprises of a cell sequence derivation method proposed by [16] that codes the GPS traces. The second step entails a forecasting method. By means of a deep learning technique named Recursive Neural Network (RNN) with a Gated Recurrent Unit (GRU), the next location of a pedestrian is predicted using the available historic cell sequence. GPS traces of visitors of a large music festival in The Netherlands, named Mysteryland, are used to train and validate the two-step procedure. The real-time forecasting capability of the new forecasting procedure are tested using time-dependent data division strategies and more elaborate cell sequence derivation methods that incorporate temporal information captured in the GPS traces.

The main contribution of this paper is the application, tuning and extension of the techniques described in [15] and [16] for the purpose of real-time pedestrian trajectory prediction at large-scale events using data gathered by means of a crowd monitoring system. In contrast to previous works, the movement behavior of the traced individuals differs severely (festival visitors instead of commuters), the GPS traces are less detailed, the granularity of the segmented network is far finer than in the original method (block size instead of neighborhoods) and the forecast is adapted to be time dependent. Parameter tuning ensured that the two techniques can now also cope with the first three differences in the characteristics in the input. The extension of the time-dependent cell sequences facilitates the forecasting of pedestrians movements for 15 min or more ahead in real-time while no historic information on those movements is available. 

This paper continues as follows. First an overview of the current literature on model- and data-driven state forecasting techniques is provided in Section 1. Section 2 will accordingly describe the crowd movement forecasting procedure. This section elaborates on the data-driven segmentation of space, cell sequence derivation methods, data division strategies, implemented forecasting models and the comparison methodology. The case-study, including site and data description, are presented in Section 3. The following Section 4 provides an overview of the results, which comprises of a benchmark of the RNN-GRU against several shallow neural networks and Markov chain models which incorporate varying degrees of memory. Finally Section 5 discusses the findings, provides preliminary conclusions regarding the applicability of the new crowd movement forecasting procedure and identifies several avenues for future research.

## 2. Contemporary Model- and Data Driven (Crowd) Forecasting Methods

In recent years, several forecasting methods have been introduced. This section provides an overview of methods that are or can potentially be used to forecast crowd movements. First, model-driven crowd movement forecasting methods are discussed. Afterwards the data-driven crowd movement forecasting methods are examined. Subsequently, data-driven traffic forecasting methods are reviewed. This section ends with a discussion of the opportunities of contemporary forecasting methods with respect to the forecasting of crowd movements at large-scale events.

### 2.1. Model-Driven Forecasting of Crowd Movements

From the 1970s onwards a number of pedestrian simulation models have been presented. In the early years predominantly microscopic multi-agent simulation models were presented, such as Cellular Automata [17], the Social Force Model [18] and the Velocity-Obstacle model [19,20]. Microscopic models compute the movements of each pedestrian in a certain space as a reaction to the presence of other objects, pedestrians and their current movement goal in a discrete or continuous fashion. The computational effort of microscopic pedestrian simulation models tends to be very high and increases rapidly when modelling large crowds. Real-time applications of microscopic pedestrian simulation models are non-existent, given their high computational effort. Yet, they are often used to perform scenario analyses of current and/or future pedestrian infrastructure designs.

More recently, multiple macroscopic crowd simulation models have been presented, which treat a pedestrian crowd as a continuous moving medium (e.g., water). While earlier versions, such as [21,22], could only model crowd movements of a single homogeneous population with one movement goal, contemporary macroscopic simulation models, for instance [23,24,25], can handle heterogeneous populations and interacting flows. Also macroscopic pedestrian simulation models have not yet been used for forecasting purposes, as they are, at this moment, not equipped to simulate the unfolding of events based on a current data-driven state estimation based on real-time data from sensors.

### 2.2. Data-Driven Forecasting of Crowd Movement

Most studies featuring the forecasting of pedestrian movements design and train models that forecast pedestrian trajectories (e.g., [26,27,28,29]). Generally, these algorithms derive the current movement behavior of a pedestrian by means of computer vision algorithms. Afterwards, a multitude of prediction techniques is used to identify the next steps of one or more pedestrians, ranging from Mixed Markov models to Recurrent Neural Networks (RNN). The prediction horizon of trajectory prediction algorithms is generally limited and the input is very detailed. This limits the applicability of these algorithms to large-scale crowd movements through an urban networks, whose movements are spread over a few hours while the real-time information regarding their movements is often spatially and temporally sparse. 

A very limited number of studies presented methods to predict the longer term movements of pedestrians, such as route and destination choice. Most of these works featured the prediction of destination sequences, which are often captured using a network of Wi-Fi sensors. Destination sequence prediction algorithms feature Bayesian approaches (e.g., [30]), dynamic discrete choice models (e.g., [31]), Markov chain models (e.g., [32]) and Hidden Markov models (e.g., [33]). One study determined the next link in a route using a link-based route measurement model that was trained by means of GPS traces [34]. 

While the prediction horizon of the trajectory prediction algorithms is too short, the destination sequence prediction algorithms could be used to predict the movements of visitors of a large-scale event. However, the presented algorithms are known for their lack of memory. As we expect that the future movements of visitors at a large-scale event are dependent on their previous movements, also the destination sequence prediction algorithms might suffice in their current form. An algorithm with long-term memory retention capabilities is sought after.

### 2.3. Data-Driven Forecasting of Traffic

In other transport-related fields, more specifically the modelling of highway and urban vehicular traffic, several interesting techniques have been introduced that can possibly be adapted to forecast crowd movements. These data-driven traffic forecasting techniques can be subdivided in three categories, being: (1) filtering techniques and stochastic models that use very limited historic information when trained, (2) time series models and machine learning algorithms that incorporate some information regarding the transition probability between states and (3) machine learning algorithms that incorporate information about the recent past of the system in real-time when computing the future state of the system.

The first group is dominated by Kalman Filter and Particle Filter applications. The Kalman Filtering technique, developed by [35], is a unified approach for the prediction of processes with a state-space representation (e.g., [36,37]). Most applications of the Kalman Filter (KF) improve the filter by adding additional knowledge regarding the forecasting problem, for example traffic engineering principles. The generic version of the Kalman Filter is a linear approximation. The Extended Kalman Filter (EKF), which is an extension of the Kalman Filter, is better equipped to also capture non-linear trends in traffic data (e.g., [38]).

The Particle Filter (PF), applied among others by [39,40], computes the posterior distribution of the states given noisy and partial observations. KFs, EKFs and PFs have a low data requirement, limited model description and low computational effort. Next to that, these filters react to local data, and can as such be categorized as techniques that incorporate very little memory regarding previous traffic states. 

Over the years, several uni- and multivariate time series models and simple neural networks have been introduced for traffic forecasting purposes, many of which incorporate the average dynamics of historic data. Most used are Gaussian Maximum Likelihood models (GML), support vector machines (SVM/SVR) and Hidden Markov Models (HMM). GML models forecast traffic using a stochastic model that combines the statistical properties of historic traffic data with real-time traffic data (e.g., [41]). This model type is simple to implement, robust and especially good at predicting global trends in traffic data, such as traffic flows. SVR is a universal learning method developed by [42]. Using the statistical properties of traffic, such as the day-to-day and within-day variation, and observational data, a statistical model is estimated. This model is accordingly used to, among other things, predict traffic volumes. Traditional SVR, introduced by [41], requires complete model training whenever new data point is added, while the online SVR (OL-SVR) can react to the current state of traffic without fully retraining the model [43,44]. HMMs forecast traffic by means of a set of pre-defined states and state transition probabilities between states. A HMM estimates the most likely sequence of traffic states [45]. This type of model assumes that the future state is independent of past realisations. As a result thereof, this model is officially memory-less. Yet, as the state transition probabilities are trained using historical traffic data, the model ‘remembers’ the average dynamics captured within the historical data. Shallow neural networks (NN) have proven to be a powerful modelling technique, which forecasts traffic using a net of interconnected linear processing elements. Neural networks are non-parametric; therefore no assumptions regarding the functional form of the underlying distribution of the data need to be made [46]. This technique can capture non-linear dependencies, but only incorporate memory regarding the average state of a traffic system. 

The last group of traffic forecasting techniques features machine learning. The ‘simplest’ version of a machine learning technique that incorporates dynamic features of the past realisations of traffic patterns is the auto-regressive integrated moving average (ARIMA) model ARIMA (e.g., [47]). This type of model forecasts future realisations of traffic flow variables based on their correlation with historic realisations, the difference between the current and historic realisations and the moving average of the most recent realisations. ARIMA models are generally trained using historic data regarding one or more features of traffic. The memory of an ARIMA is limited to a certain period in time. More sophisticated machine learning techniques, such as recurrent neural networks (RNN) with and/or without the long short-term memory (RNN-LSTM) or gated recurrent units (RNN-GRU), have been introduced to overcome some of the shortcomings of the shallow neural networks, namely their lack of memory. The inclusion of memory allows RNN networks to incorporate the ordering of events. That is, an RNN can distinguish between the arrays [1,2,3,4] and [4,2,3,1] even though the numbers within the two arrays are similar. In recent years, the popularity of RNN-based solutions for the prediction of macroscopic as well as microscopic properties of traffic has increased hand over hand (e.g., [15,48,49,50]). Comparisons of the performance of RNN-based solutions to shallow neural network and ARIMA models illustrate that RNN-based solutions are better equipped to predict traffic properties [48,49]. Moreover, the performance difference between the two main RNN-based solutions is negligible. 

The dependencies in traffic are highly non-linear and the current movement pattern of an individual is related to its past actions. Consequently, to predict crowd movements a forecasting method is needed that can handle non-linear dependencies and has a memory. Table 1 presents and overview of the methods discussed in the previous paragraphs, and has categorised them with respect to these two properties. Given the properties of traffic, it can be concluded that we need to apply a technique that falls in the lower right corner of the table.

## 3. Methodology

The review of contemporary works on the forecasting of crowds and traffic illustrates that especially recurrent neural networks are equipped to forecast crowd movements. Yet, up to this moment a RNN has never been applied for this purpose. Considering the work on traffic forecasting methods, the work of [15] is most in line with the objective of this study, namely the method forecasts a sequence of locations visited by a vehicle in a large street network. A great benefit of the approach presented by [15] is the very limited computational effort involved in data filtering and training. The use of smartly chosen cells instead of a directed graph consisting of nodes and links diminishes the computational effort greatly, as map-matching of GPS traces to the network is circumvented. Moreover, the use of a small number of cells that automatically adopt the structure of the transportation network, allows for a reasonably valid and logical discretization of the network.

Yet, the approach cannot be adopted without changes as the movement patterns of visitors at event terrains differ in three ways from vehicular traffic during morning rush hour. Firstly, the temporal dimension of the movement pattern differs. That is, movement patterns at events are dependent on the stage programming which changes quickly throughout the entire day. Specifically at the beginning and end of programmed shows high flows arise that develop quickly and disappear just as fast. In order to catch these sudden increases and decreases of flow rates predictions at 5 min intervals instead of hours are needed from opening till closing time. Secondly, activities do not only occur at the nodes of a network, but also on the links in between. As a result, in contrast to the original approach, the crowd movement forecasting approach should be capable to predict stay-in-place traffic as well as traffic that moves through the corridors of the network. Thirdly, pedestrians at events do not disappear from the traffic network when they reach their location. Consequently, no identification of the end of a cell sequence is necessary. 

As a result of the differences in the traffic and crowd movement patterns, adaptations to the original approach presented by [15] are needed. The following section will detail the adapted forecasting approach. In essence, the approach consists of three steps, being: (1) the segmentation of space, (2) the identification of cell sequences and (3) the training of the RNN-GRU. As in this research the forecasting results of the RNN-GRU will be compared using six comparison models, namely three shallow neural networks and three Markov chain model that incorporate varying degrees of memory. Furthermore, this research wants to test how valid the predictions by all seven models are, and whether the best forecasting model is capable of performing real-time predictions while an event is ongoing. Therefore, the research methodology also includes a cross- validation step and a test in which several additional data division methods and sequencing strategies are tested. 

**Figure 1:**
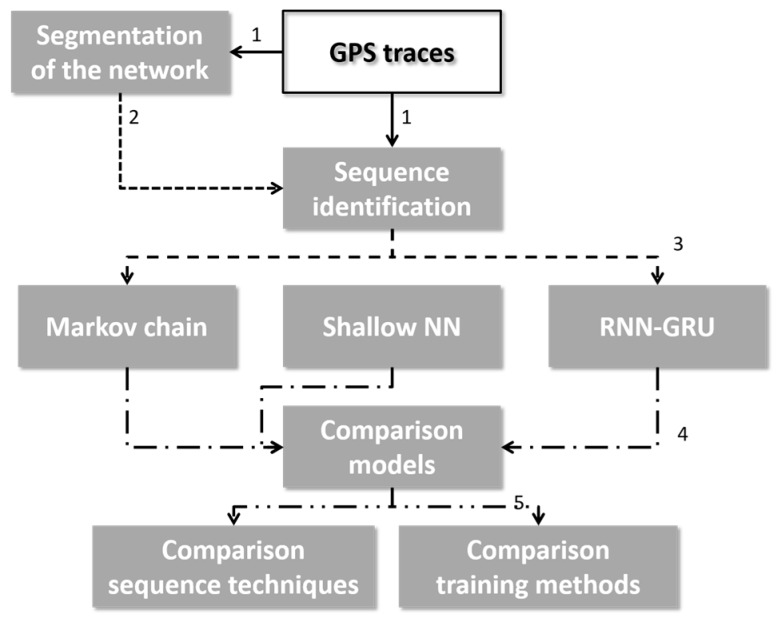
Visualization of the research methodology, where the numbers identify the types of data that are the input of the modules. (1) GPS traces, (2) set of Voronoi cells, (3) Cell sequences, (4) result of model prediction, (5) optimal type of sequence prediction model.

The complete research methodology is visualized in Figure 1. The following paragraphs will elaborate on the steps of the research methodology. The first Section 3.1 details the data-driven segmentation of space by means of the segmentation algorithm presented in [16]. The second Section 3.2 elaborates on the filtering of the cell sequences in order to include the temporal element into the method of [15]. Section 3.3 details the data division methods, which are used in order to test the real-time estimation capabilities of the best model. The following Section 3.4 describe the design and training the three model types. The last Section 3.5 details the methodology of comparing the models, including the metrics used to determine the best model.

### 3.1. Data-Driven Segmentation of Space

In order to capture the movements over the network, first the raw GPS traces are translated into sequences of cells that have been visited by a pedestrian that are ordered by time. This has as advantage that each GPS trace is simplified into an array that contains only main movement features of the pedestrian. The discretized sequence of cells can be handled by an RNN. Any segmentation of space based on a purely mathematical description would be a behavioral assumption on the researchers’ side, which has as a disadvantage that the cell structure does not follow the travel behavior of the traffic. By allowing the data to show us the best segmentation of the network, we ensure that the especially the most heavily travelled corridors in the network are correctly segmented. More specifically, this research adopts the data-driven space segmentation technique presented in [16], which is an adapted version of the technique originally presented by [51]. See Algorithm 1 for a summary of this technique. 

Essential to this method is the choice of the order in which the seed points are fed to the algorithm. When seed points are randomly drawn from the data set, the structure of the segmented space changes with each attempt. In order to create a stable and behaviorally valid segmentation of space, the set of seed points is ordered based on density before are fed to the algorithm. This ensures that the most heavily travelled corridors in the traffic network are optimally represented by the cell structure. Yet, a sensitivity analysis is necessary to ensure a stable space segmentation solution is derived, which is not depending heavily on the seed points feeding order. 

In this research all coordinates $\vec{x_{p}}\left(t\right)$ that are part of the full set of GPS traces are used to feed the data-driven space segmentation algorithm. Given that GPS traces develop over time, this might not be completely realistic. However, given that the focus of this paper is on the development of the forecasting method, it essential to use a static fully defined segmented network as the basis for all estimated forecasting models. In reality, when using this algorithm on the fly, the network would become more defined during the runtime of the forecasting method. The effect of dynamic changes of the network are severe, as recompiling the paths and retraining of the forecasting model are essential whenever the segmentation of the network changes. More research is needed into methods to determine at which moments in time recompiling the segmented network is most opportune. Moreover, depending on the order in which locations are visited by the crowd influences the parts of the segmented network that are best defined. The current usage of the full set of GPS traces eliminates dynamic shifts in network definition. Also with respect to this issue, more research is advised.
**Algorithm 1**: Description of the data-driven space segmentation algorithm presented by [13], which segments space into a set of cells and derives the sequence of cells visited by each of the pedestrians at the event.**Input**: Set of seed points $X\;=\left\{\vec{x_{p}}\left(t\right)\right\}$, desired maximum cell radius *γ* **Output**: Raw sequences of cells $S_{p}^{raw}$ with the cell identification number $s_{p}\left(t\right)$ for each point $\;\vec{x_{p}}\left(t\right)$ in every GPS trace $X_{p}\left(t\right)$
Initialize C={}For each seed point $\vec{x_{p}}\left(t\right)$
Find the closest cell $c_{n}$ in C, where the distance between $\vec{x_{p}}\left(t\right)$ and $c_{n}$’s centroid is less than or equal to *γ* and $c_{n}$’s centroid is closer to $\vec{x_{p}}\left(t\right)$ than any other cell centroids in CIf such *c* is not found, create a new cell C and assign $\vec{x_{p}}\left(t\right)$ to $c_{n+1}$. Add $c_{n+1}$ to CAssign s to c and update c’s centroid as the middle point of the current collection of members of the set of seed points that is currently assigned to cFor each cell $c_{n}$ in C, remove all member points while keeping its centroidFor each point $\vec{x_{p}}\left(t\right)$ in X, find the closest cell $c_{n}$ in C and set $s_{p}\left(t\right)=c_{n}$.
Where $c_{n}$ is a cell in set C with index *n*, $\vec{x_{p}}\left(t\right)$ a seed point of the set $\vec{X}$ that represents the coordinates of pedestrian *p* at time *t*.

**Figure 2:**
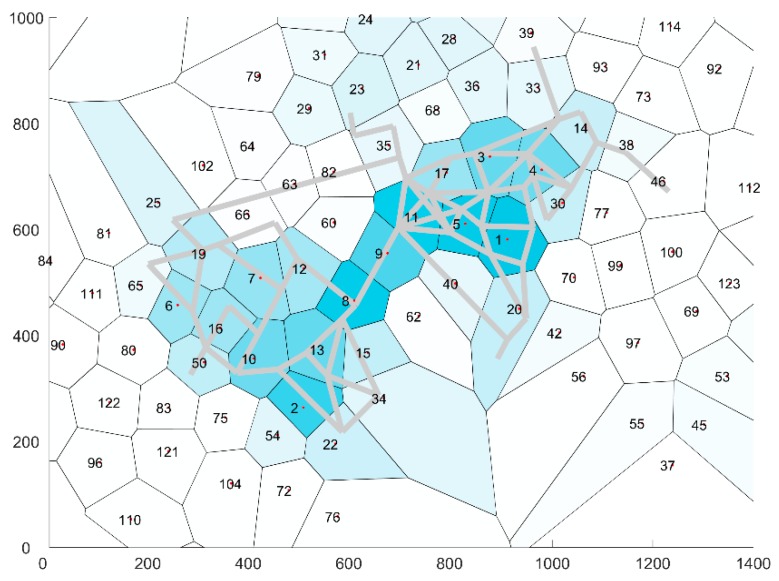
Number of hits per cell distributed over the area, where the grey lines identify the network of the Mysteryland festival area in 2017. Darker coloring of the cells represents a higher number of hits.

In essence, this algorithm derives a Voronoi diagram with cells which have a limited radius. The cell centroids of each Voronoi cell are accordingly used to partition the network in distinct zones, determine the boundaries between these zones and allocate all point of a GPS trace to each zone, hereafter the zones are named cells. The partitioned network is shown in Figure 2. The desired maximum cell radius is set to γ=100 m. The authors have tried several settings of the maximum cell radius and found that this setting provides the best forecasting results. The current setting of the maximum cell radius is a balancing act between largest radius that can practically be adopted for crowd management purposes in urban networks, the smallest radius for which the GPS traces will provide viable results and the steeply increasing burden of model estimation whenever a lower maximum cell radius is adopted. Given that a cell of at maximum 31,415 m^2^ is quite a large area, the current setting limits the use of this forecasting approach to pedestrian infrastructures that consist of a network of large corridors and open areas. Important to note is that these settings cannot be used to indicate local crowd management issues, such as an increase in density or flow in a small path way. This setting relies on crowd managers to make the translation of global trends in the movement behavior to very specific local issues such as overcrowding.

At free flow speed pedestrians can traverse 390 m per 5 min, which is more than the maximum cell radius. Moreover, not all cell boundaries will be limited by the maximum cell radius. That is, cells with a smaller cell radius will exist. These cells are generally located in-between cells with a high density of seed points. In both cases, pedestrians can cross multiple cells within the prediction horizon. Consequently, pedestrians might not be registered at intermediate cells. This limits the realism of the forecasted paths of each pedestrian. Yet, given that crowd management officials require fairly detailed forecasts in order to determine their intervention strategy, the authors deem the detail of the actual aggregate forecast more important than forecasting a realistic viable path for each pedestrian.

### 3.2. Identification of Cell Sequences

The result of the previous step is a cell sequence ordered by time for each GPS trace. These cell sequences need to be translated into cell sequences that can be handled by the forecasting model. There are several different ways to translate the cell sequences, which are more or less time independent and/or a valid representation of the behavior of the individuals. Underneath three distinct methods are detailed, namely a consecutive hit method, the first hit method and the longest stay method. 

**Figure 3:**
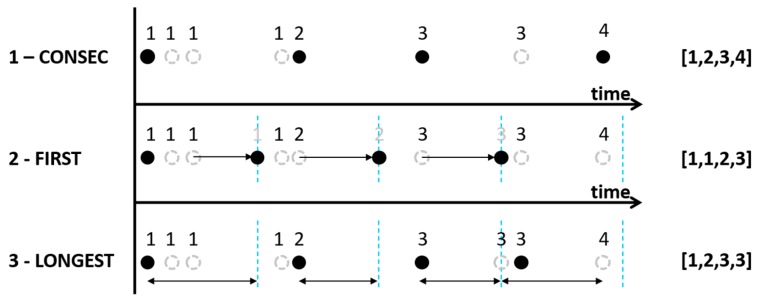
Methods to filter the raw cell sequences, where dashed open cells are all points in the GPS trace, the closed cells are the cells selected according to each method, and the resulting raw cell sequences are identified as [x,x,x,x].

Using the consecutive hit method, these the raw cell sequences are filtered to only contain dissimilar consecutive cells. Basically, this sequencing method determines only the sequence of cells that has been touched by the GPS trace (hereafter named *‘Consec’*). Because GPS points within a GPS trace are not temporally equally divided, the cell sequences are time independent (see method 1 in Figure 3 and Algorithm 2). That is, the cell sequence identifies where pedestrians went, but it is not clear how long they stayed in each cell.
**Algorithm 2**: Description of the consecutive hit algorithm, which translates the raw cell sequence to a cell sequence of consecutive visited cells**Input**: Raw sequences of cells $S_{p}^{raw}$ **Output**: Filtered sequences of cells $S_{p}^{filt}$For each cell sequence $S_{p}^{raw}$
Set $S_{p}^{filt}=\{\}$For each record $s_{p}\left(t\right)$ in cell sequence $S_{p}^{raw}$, determine $s_{p}\left(t-1\right)$ == $s_{p}\left(t\right)$
If $s_{p}\left(t-1\right)$ == $s_{p}\left(t\right)$, $s_{p}\left(t\right)$ is added to the cell sequence $S_{p}^{filt}$If $s_{p}\left(t-1\right)$ == $s_{p}\left(t\right)$, do nothing.
Where $S_{p}^{raw}$, $S_{p}^{filt}$ are the raw and filtered cell sequences and of pedestrian *p*, consisting of records $s_{p}\left(t\right)$.

However, temporal information is essential to create a forecast of their movements at an event. Therefore also two methods are adopted that incorporate a temporal dimension in the cell sequences. The first method divides time in equal time periods of 5 min. Accordingly, for each time interval the cells are selected that are closest to the beginning of a time period (hereafter named ‘*First’* - see Method 2 in Figure 3 and Algorithm 3). In general, the last cell visited during the previous time period is identified as the current cell. In cases where pedestrians are standing still in one cell the previous cell is similar to the future cell, so no differences are found. However, in cases where a pedestrian is moving, historic location information is favored over future location information.
**Algorithm 3:** Description of the first hit algorithm, which translates the raw cell sequence to a cell sequence of cells that were first visited in each time period.**Input**: Raw sequences of cells $S_{p}^{raw}$ **Output**: Filtered sequences of cells $S_{p}^{filt}$2.Create the set K3.Set $S_{p}^{filt}=\{\}$4.For each cell sequence $S_{p}^{raw}$
For each k in K
Identify the cell $s_{p}\left(t\right)$ nearest to timestep kSet $s_{p}\left(k\right)=s_{p}\left(t\right)$Add $s_{p}\left(k\right)$ to $S_{p}^{filt}$
Where $S_{p}^{raw}$, $S_{p}^{filt}$ are the raw and filtered cell sequences and of pedestrian *p*, consisting of records $s_{p}\left(t\right)$, K is a set of equally spaced timesteps k that indicate the beginning of a new time period

The second method selects the cell whose visit duration has been longest within each 5 min interval (hereafter named ‘*Longest’* – see Method 3 in Figure 3 and Algorithm 4).
**Algorithm 4:** Description of the longest cell duration algorithm, which translates the raw cell sequence to a cell sequence of cells that was visited the longest during each time period**Input**: Raw sequences of cells $S_{p}^{raw}$ **Output**: Filtered sequences of cells $S_{p}^{filt}$Create the set KSet $S_{p}^{filt}=\{\}$For each cell sequence $S_{p}^{raw}$
For each k in K
Set D, which consist of n 0’sDetermine the time spend in each cell $d_{n}$ during time period k≤t<k+1Identify the cell $s_{p}\left(t\right)$ for which $d_{n}=\underset{}{\max}D$Set $s_{p}\left(k\right)=s_{p}\left(t\right)$Add $s_{p}\left(k\right)$ to $S_{p}^{filt}$
Where $S_{p}^{raw}$, $S_{p}^{filt}$ are the raw and filtered cell sequences and of pedestrian *p*, consisting of records $s_{p}\left(t\right)$, K is a set of equally spaced timesteps k that indicate the beginning of a new time period

Here, the time interval is the time between the last time a GPS trace was detected in another cell and the first moment in time that the GPS trace was detected in the current cell. If these moments in time are earlier and/or later than the 5 min interval, the boundaries of the interval are taken as the start and/or end of the time period. Consequently, the stay duration can never be larger than 5 min for each time interval. 

To compare the model types only Method 1 is used, which is similar to the cell sequence identification method applied by [15]. Methods 2 and 3 are accordingly adopted to determine whether the best method is also capable of performing real-time predictions. 

### 3.3. Division between Training and Validation Data

There are multiple ways to divide the total number of cell sequences between a training and a validation dataset. One of which, is to randomly sample a certain percentage of sequences from the total set to serve as cross-validation set, while the remaining sequences serve as training set (hereafter identified as ‘*Random*’). The advantage of this division method is that all types of sequencing behaviors are including in the cell sequences used for training as well as testing.

If one, however, wants to use the cell sequence prediction algorithm to predict the visitors’ movements while the event is ongoing, one needs to train the model while the event is ongoing. One way of doing so, is to use the data of the first day to train in order to predict cell sequences on day two, hereafter named ‘Sat/Sun’. Similar to the Random division method, the training data contains cell sequences that represent the crowds’ movements during the morning, afternoon and evening. In contrast, only the data of the Saturday is used for training. As such, this training method only is expected to work if the cell sequencing behavior of the visitors is similar on both days.

Another way to split the dataset based on time of day. This method uses the first few hours of a day to train the model and predict the movements of the visitors later on the day. To mimic this type of training, the sequences are split based on the time of day. That is, the data of the first six hours of the day (10:00–16:00) are used to predict the cell sequences occurring during the last eight hours of the day (16:00–24:00). This dataset division method is hereafter named ‘*M/E*’. In contrast to the Random and Sat/Sun division methods, this method is only expected to work if the cell sequencing behavior of the visitors in the morning and beginning of the afternoon is similar to the late afternoon and evening.

In order to determine the best model type the Random division method is chosen, which contains all behaviors in both the training and testing data sets. This allows us to test whether the model types can capture all crowd movements. Afterwards, the best model type is trained with the other training and test datasets to see whether the chosen model structure is also able to be used for real-time predictions. 

### 3.4. Tested Models

The sequence of cells traversed by a visitor is modelled using four types of models, namely a Recursive Neural Network with a Gated Recurrent Unit (RNN-GRU), a shallow neural network and a first-order Markov chain. Underneath the design and training of the four models is elaborated upon briefly. 

The RNN-GRU is a neural network which loops information through a cell, which allows information to persist. In case of a GRU, an update and reset gate determine what information is passed to the output. The RNN-GRU used in this research consists of 150 hidden nodes. The model is trained in batches of 100 records and 10 epochs. The Adam optimization algorithm is used to train the RNN-GRU [52].

Besides the RNN-GRU three simple neural network are trained, with no memory (NN-1), a memory of order one (NN-2) and a memory of order two (NN-3). This model consists of an input layer of one, two or three times the number of cells as nodes, one hidden layer of 150 nodes and one output layer with a length of the number of cells. The input consists of one, two or three one-hot variables pasted behind each other. The output is a one-hot variable. These pattern recognition networks are all trained in Matlab using a resilient backpropagation algorithm with a learning rate of 0.01.

Similar to the shallow neural networks, three Markov chains are estimated that incorporate incrementally more memory, namely a first-order (MC-1), second-order (MC-2) and third-order (MC-3) Markov chain. Depending on the order only the last *m* visited cells are used to determine the transition matrix. Accordingly, the cell with the highest probability is adopted as the next cell in the sequence.

### 3.5. Comparison of the Trained Models Featuring Crowd Movement Prediction

The forecast model should have two important features, namely:

1. The model correctly predicts the right next cell in the sequence,
2. or in case the wrong cell is identified, this ‘wrong’ cell is located nearby the cell that would have been correct.

The first feature is quantified using the error rate of the model (seq. 1), where $N_{s=l}{}^{m}$ is the number of outputs that is predicted correctly by model *m* and $N_{s\neq l}{}^{m}$ the number of outputs that are not predicted correctly. Here, a subscripted *s* identifies one label in the simulated cell sequences and *l* one label in the actual recorded cell sequences:

(1) $$E^{m}=\frac{N_{s=l}{}^{m}}{N_{s=l}{}^{m}+N_{s\neq l}{}^{m}}$$

As the three types of models have distinct memory-retention structures, the error rate might differ depending on the length of a sequence that is already known. Therefore, three error-rates are computed for the second, fifth and twentieth cell in a sequence. Here, the hypothesis is that the RNN-GRU model excels in the correct prediction of the next cell at further on in the sequences as the memory of this type of model is most extensive.

The second characteristic is tested using the average error distance of each cell sequence prediction model. This is operationalized as the mean absolute Euclidian distance between the location of the chosen cell and the location of the predicted cell. Here, $N_{c}$ represents the total number of data points that is estimated, ${\vec{x}}_{l}{}^{m}$ the location of the centroid of the recorded cell and ${\vec{x}}_{s}{}^{m}$ the location of the centroid of the predicted cell:

(2) $$D^{m}=\frac{1}{N_{c}}\|{\vec{x}}_{l}{}^{m}-{\vec{x}}_{s}{}^{m}\|$$

It is expected that a perfect cell sequence prediction model has an error distance of 0.

## 4. Case Study Description

Data from a case study is used to train and test the adapted approach, namely data from the dance festival Mysteryland. The following paragraph will first introduce the festival (see Section 4.1). Accordingly, the characteristics of the GPS traces are discussed in Section 4.2. The following Section 4.3 elaborates on the filtering of the GPS traces. The last part of this Section 4.4 presents the characteristics of the cell sequences that serve as input for the cell sequence prediction models.

### 4.1. Mysteryland 2017—The Netherlands

Every year a large dance festival is held at the Floriade terrain near Amsterdam in The Netherlands. Within this area of approximately 600,000 m^2^ in total almost 100,000 visitors enjoy different sorts of dance music for one or two days. In 2017, this event was held on August 26th and 27th, two days with almost no rain, a temperature of 24 °C and limited cloud cover. The population of the event is fairly young (18–35 years old). The visitors generally moved around in groups. Furthermore, both genders were equally represented at the festival terrain. Reference [6] illustrates that the activity choice behavior of the crowd showed two distinctive patterns. Either visitors walked around visiting a number of stages with a distinct music taste or visitors remained at their preferred stage for large parts of the day. In order to manage these crowds effectively, it is essential to predict during the day at what moment in time the crowd is moving and the route that they take from one stage to the next.

### 4.2. Characteristics of GPS Traces

TU Delft has been developing a crowd monitoring system for some time. The input of this CMS is very diverse, ranging from automatic counting data to GPS traces. Mysteryland is one of the (music) events which are used to test new data sources, data fusion algorithms and forecasting algorithms. In previous years, the use of GPS traces as an input to the CMS has been studied by [6] and [7], but deemed too labour intensive to be a viable data source for the CMS. However, a smartphone application of Woov opened up a different, less labour intensive, way to gather GPS traces in real-time. The aim of the joint project between TU Delft and Woov at Mysteryland was to determine whether the GPS traces gathered by the smartphone application of Woov had enough explanatory power to be used for the real-time monitoring and online forecasting of crowd movements at large-scale events. 

The company Woov has built a smartphone application for the Mysteryland event which informs its users regarding the programming, the upcoming events and location of facilities (e.g., toilets, food, first aid) on the terrain. More importantly, this application allows its users to determine the current location of their friends on the terrain and provides the users all essential information to find their friends. Friends are, according to the definition of Woov, individuals that granted another specific person access to their location-information for the duration of the event within the region where the event takes place. In order to provide location information regarding a friends’ location, the smartphone application records the GPS locations of all individuals that use this app (with their consent) and some characteristics of each person (e.g., age, gender, network of friends). The application of Woov asks the location of all smartphones connected to their application, which they impersonalize and save in their database for short-term usage. The GPS data consists of latitude, longitude, timestamp, accuracy, id number of the person, age and gender. The services provided by the application (i.e., finding friends, getting information regarding facilities at the terrain and receiving instructions from the event organiser in real-time) are the only incentives that users get for providing their location information and socio-demographic information. The terms of use of the smartphone application details what the company Woov is allowed to do with the data provided by the users. By accepting the terms of use, the users accepts that their location information is used for several purposes. 

Only the anonymized GPS data, and some metadata thereof, were shared with TU Delft, as a part of a joint project after the unfolding of the event. Each person in the shared GPS data is identified by an id number. Consequently, the researchers do not know to whom a certain trace belongs. The location-information of all users of the application is used in this research.

The dataset of Woov captured location data on 9,748 distinct individuals at Mysteryland. After filtering (see the following section for the details), 13,461 continuous trajectories. As the GPS position is determined using a number of complex algorithms, the accuracy of the measurements is reasonably high (i.e., ±10 m).

### 4.3. Cleaning of the GPS Traces

The raw GPS traces include a lot of noise. The first filter step is to detect outliers and discard those. Sudden changes in speed and direction are used to determine outlier data points. In order to allow state estimation, frequent location information of one individual is essential within the Mysteryland event terrain. That is, enough data points to allow one to determine the full route of a visitor through the network and have an estimate of the speeds that were travelled by the pedestrian on each link. This translates into three filtering criteria, namely: (1) all data points outside the Mysteryland event terrain are discarded, (2) if the absolute speed between two consecutive data points is larger than 5 m/s, these points are discarded, (3) if a trajectory contains a gap which is larger than 10 min, the trajectory is split into two separate parts. 

### 4.4. Derived Cell Sequences

The average time interval of a GPS trace of one visitor was 17.8 h during which an average distance of 5.4 km was covered by the visitor. These traces are accordingly translated into a set of cells by the method described in Section 3.1. Accordingly the traces are translated in a sequence of cells. Figure 2 illustrates the number of hits per cell. Most hits are located near the three largest music stages, which are located in cells 1, 2 and 8. 

**Figure 4:**
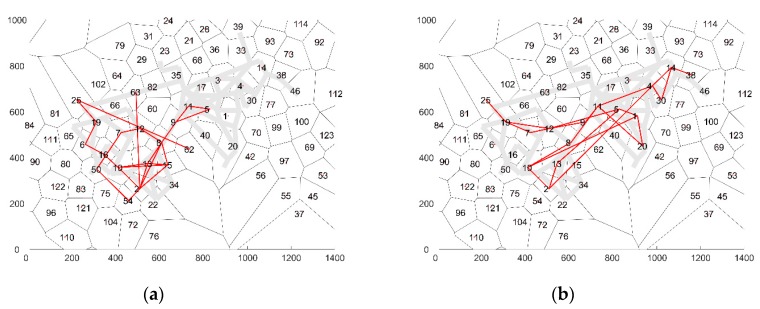
Two examples of cell sequences, where (**a**) displays a trajectory that only visited one side of the terrain, (**b**) presents a trajectory that covered the entire terrain.

The average number of cells in each sequence is 120.7. Two example cell sequences are visualized in Figure 4. As one can see, the consecutive cells in a sequence are not necessarily adjoining cells. The reason for this artefact in the cell sequences is that the temporal resolution of the GPS traces is relatively low (5–10 min), which is sparser than the maximum cell radius. Therefore, as the aim is to predict the cell sequence behavior of the visitors, the cell sequence prediction models should be allowed to skip adjoining cells as well.

**Figure 5:**
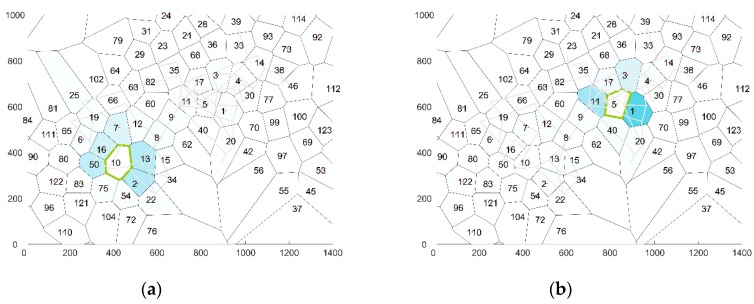
Visualization of the average transition probabilities towards other cells for (**a**): cell 10 and (**b**) cell 50.

An analysis of the average transition probabilities for a specific cell shows that in general adjoining cells have a relative higher transition probability than cells further afield. At the same time, in some cases the transition probabilities for all adjoining cells are almost similar (see Figure 5a), while in other cases, one adjoining cell is favored over the other cells (see Figure 5a). The best model is expected to recreate the transition probabilities depicted above.

## 5. Forecasting Results

In total, seven models have been estimated. Table 2 presents a summary of the validation results for each of the models. In the table the error rates and average error distance are mentioned for the 1st, 5th and 20th prediction of the sequences. Here, the Nth prediction identifies the prediction of the Nth visited cell in a particular cell sequence given that all previous locations (1 untill N-1th) are specified as input. We expect that the models that incorporate a memory of previous steps predict the cell location of steps later in the sequence better than the location of steps early on in the sequence. 

The table illustrates that the Markov chain and RNN-GRU model structures are slightly better at predicting the next cell at the end of a sequence, than the beginning of a sequence. Contrary to our expectation, the difference in the error rates for the 1st, 5th and 20th predictions of a particular model vary only slightly. This suggests that only the previous one or two visited cells influence the current cell visited. At the same time, the fact that the RNN-GRU outperforms the Markov Chain models at any stage of a cell sequence. RNN-GRU’s includes more complex conditional probability distributions to model transitions between cells. Therefore, the outperformance suggests that long-term dependencies exist in the movement behavior of the visitors that are more easily captured by the RNN-GRU model.

The table illustrates that the two goodness-of-fit measures indicate two distinct model structures. That is, the RNN-GRU predicts the next cell most often correctly. We expect the low error rate to be the consequence of the incorporation of the memory of earlier steps in the sequence. However, if the wrong next cell is predicted, the Markov chain is more likely to predict another next cell near the cell that was actually visited. Given that the 2nd-order Markov chains have a very high error rate and the average error distance of the RNN-GRU is almost similar to the error distance of the 3rd order Markov chain, the RNN-GRU is chosen to be the best model amongst the tested models to predict the cell sequence behavior of the visitors at Mysteryland. The RNN-GRU model predicts the correct next cell in approximately 82.3% of the time. Besides that, when the model predicts the wrong location, in many cases, a cell adjoining the correct cell is identified.

However, these seven model structures have been trained in a very specific manner. The use of a training set with a random drawn selection of cell sequencing featuring crowd movements on both days and all hours of the event does limit the usage of the model for real-time prediction purposes. Moreover, the current cell sequencing method is time independent, which makes a translation from cell sequences to network flows very difficult. In order to determine whether the current model structure could be used for real-time prediction purposes, the RNN-GRU has also been trained using two other training methods (i.e., Sat/Sun and M/E) and two other sequencing methods (First and Longest). 

The results related to all combinations of the three training methods and three sequencing methods are displayed in Table 3. Surprisingly, the models trained by means of the datasets that were divided by means of the Sat/Sun and M/E division methods outperform the Random division method. This might be because the GPS traces are slightly different on both days. As models that are trained on data divided by means of the Random division method are trained on data from both days, they are slightly more generic than models trained on data from either of the two days. This lack of specification leads to a slight reduction of the fit. Yet, the differences in error rate and average distance error are fairly small. Consequently, this study concludes that the RNN-GRU can be used for real-time prediction purposes. 

Table 3 also indicates that the error rate and average error distance decrease when one of the two other cell sequencing methods (i.e., First and Longest) is used. In general, the error rate is best when using the cell sequencing method First. Moreover, the average error distance is lowest when using this cell sequencing technique. Therefore, this model is identified as the best model amongst the tested models to predict the cell sequencing behavior of visitors of Mysteryland.

## 6. Conclusions

This paper has tuned and enhanced an existing data-driven urban traffic forecasting technique presented by [15] and [16] to predict pedestrian movements at large-scale event terrains. Parameter tuning of the cell sequencing technique and cell sequence filtering algorithm ensured that the forecasting model can now also cope with the first three differences in the characteristics in the input. The extension of the time-dependent cell sequences facilitates the forecasting of pedestrians movements for 15 min or more ahead in real-time while no historic information on those movements is available. Using the cell derivation method first proposed by [16] GPS traces of pedestrian movements have been translated into individual cell sequences. These cell sequences were accordingly used to train and validate seven distinct model structures, namely three shallow neural networks, three Markov chain models of different orders and a RNN-GRU. The RNN-GRU model outperformed all other model structures. Accordingly, the RNN-GRU model was trained using three types of data division methods and three types of cell sequencing techniques. The results illustrated that the RNN-GRU preserves its predictive power when a limited amount of data is used from the first few hours of a multi-day event. Moreover, the error in the prediction decreases when the length of the stay in a cell is taken into account. 

Consequently, this research concludes that using cell sequence data and a RNN-GRU crowd movements at large-scale events can be predicted without first map-matching the GPS trajectories. Moreover, the features of the crowd movements at these terrains allow this type of network to be trained while the event is ongoing. Consequently, by means of the cell sequence data the RNN-GRU can be trained and applied for real-time crowd movement prediction purposes.

This paper has tested a specific set of model types for the purpose of cell sequence prediction. The RNN-GRU is known for its good memory retention qualities and the fact that neural networks can cope very well with the non-linear dependencies in traffic. However, the field of deep learning progresses rapidly and more types of deep learning networks with interesting features have seen the light. LSTM networks, and other RNNs with a more complex design, might improve the goodness-of-fit even further.

Moreover, this work determines how to best predict the sequence of cells traversed by one individual in order to predict the aggregate features of the crowd, such as flow and density. Noise might be introduced in the process of aggregating the individual predictions. Therefore, it is essential to also study methods that can directly estimate the characteristics of the crowd movement at an aggregate level. 

Lastly, the data of one large-scale event in The Netherlands is used to train the RNN-GRU. At this moment in time, it is unclear to what extent the features of this event, such as network size, stage programming, visitor type, influence the goodness-of-fit of this recurrent neural network. In order to determine the general applicability of a RNN-GRU for cell sequence prediction at large-scale events more research is needed.

## Figures and Tables

**Table 1:** Categorization of mathematical techniques for traffic forecasting.

	Memory Regarding Average Reaction to State of the System	Memory Regarding Average State of the System	Memory Regarding the Dynamic State Transitions of the System
Linear	Kalman Filter (KF)		ARIMA
Non-linearity	Extended Kalman filter (EKF) Particle Filter (PF) Gaussian Maximum Likelihood (GML)	Hidden Markov Model (HMM) Neural Network (NN) Support Vector Machine (SVM)	Recurrent neural network (RNN) Recurrent neural network with gated recurrent unit (RNN-GRU) Recurrent neural network with long short term memory (RNN LSTM)

**Table 2:** Summary of cross-validation results for distinct model structures, where the bold numbers identify the best model according to the goodness-of-fit metrics.

Model Network	Division Method	$\mathrm{Error}\;\mathrm{Rate}\;\left(E^{m}\right)$			$\mathrm{Average}\;\mathrm{Error}\;\mathrm{Distance}\;(D^{m})$		
		1	5	20	1	5	20
Shallow neural network	1st order	0.9780	0.9427	0.9492	366.52	309.38	322.64
Shallow neural network	2nd order	0.9468	0.9420	0.9535	469.34	363.64	305.14
Shallow neural network	3rd order	0.9604	0.9752	0.9722	451.42	456.68	464.93
Markov chain	1st order	0.7756	0.7077	0.7204	170.82	150.82	150.95
Markov chain	2nd order	0.6197	0.5786	0.5705	**135.73**	**128.69**	**121.78**
Markov chain	3rd order	0.6589	0.6576	0.6227	160.25	155.29	131.93
RNN-GRU	Inf.	**0.2772**	**0.3436**	**0.2676**	136.42	167.87	124.49

**Table 3:** Summary of cross-validation results for distinct sequencing methods and divisions between training and testing data sets.

Model Network	Seq. Method	Division Method	$\mathrm{Error}\;\mathrm{Rate}\;\left(E^{m}\right)$			$\mathrm{Average}\;\mathrm{Error}\;\mathrm{Distance}\;(D^{m})$		
			1	5	20	1	5	20
RNN-GRU	Consec	Sat/Sun	0.2217	0.2411	0.2396	168.27	160.39	173.98
RNN-GRU	Consec	M/E	0.3532	0.3272	0.3648	131.96	144.64	131.57
RNN-GRU	Consec	Random	0.2798	0.3010	0.2698	106.57	117.56	112.96
RNN-GRU	First	Sat/Sun	0.2229	0.1862	0.1840	103.43	79.59	83.64
RNN-GRU	First	M/E	0.2043	0.2135	0.2186	87.64	96.64	96.68
RNN-GRU	First	Random	0.2909	0.2586	0.2450	128.44	108.41	97.30
RNN-GRU	Longest	Sat/Sun	0.1859	0.2245	0.2245	85.18	101.07	93.99
RNN-GRU	Longest	M/E	0.1952	0.1790	0.2180	85.27	81.89	94.67
RNN-GRU	Longest	Random	0.2302	0.2332	0.2372	107.21	102.53	102.63
